# Genetic diversity and population structure assessment using molecular markers and SPAR approach in *Illicium griffithii*, a medicinally important endangered species of Northeast India

**DOI:** 10.1186/s43141-021-00211-5

**Published:** 2021-08-10

**Authors:** Rajib Borah, Atanu Bhattacharjee, Satyawada Rama Rao, Vineet Kumar, Pradeep Sharma, Krishna Upadhaya, Hiranjit Choudhury

**Affiliations:** 1 Department of Basic Sciences and Social Sciences, School of Technology, North-Eastern Hill University, Shillong, Meghalaya 793022 India; 2 Department of Biotechnology and Bioinformatics, School of Life Sciences, North-Eastern Hill University, Shillong, Meghalaya 793022 India; 3 Chemistry and Bioprospecting Division, Forest Research Institute, Indian Council of Forestry Research & Education, Dehradun, Uttarakhand 248006 India

**Keywords:** *Illicium griffithii*, Markers, Shikimic acid, Oseltamivir, Genetic diversity, SPAR

## Abstract

**Background:**

*Illicium griffithii* is an aromatic medicinal tree species that has been listed in the IUCN Red List as an endangered species. Dried seed pods of *I*. *griffithii* have a good market potential in the spices and pharmaceutical industries. Fruits are the potential source of shikimic acid and used for the production of oseltamivir (a drug against bird flu). However, in recent years, unscientific harvesting and rampant exploitation of the species has caused a negative and adverse effect on its natural population. Proper knowledge of genetic diversity and population structure is crucial to understand the population dynamics, adaptation, and evolutionary pattern of a particular species for conservation. It was from this view point that the present study was undertaken so as to compare the various types of DNA-based molecular markers namely RAPD, ISSR, DAMD, and SCoT by their efficiency and SPAR approach to evaluate the genetic diversity of *I*. *griffithii* as well as to analyze population genetic structure for conservation purpose.

**Result:**

A total of 250 discernible bands were generated with 246 bands (98.40 %) being polymorphic in nature. All the primers in combination gave a mean polymorphic information content (PIC) of 0.81 and Rp value (resolving power) of 4.32. Nei’s, Gst, and AMOVA analysis showed similar values of genetic differentiation among populations (Gst = 0.396, F_ST_ = 0.30, respectively), revealing a low level of genetic differentiation among the eight sampled populations. *I*. *griffithii* with an estimated gene flow value of Nm = 0.761 was significantly low among populations. Clustering pattern obtained with Bayesian structure and PCoA diagram revealed that intermixing of genetic material across populations is only possible when the populations lie close to each other. This is further validated with UPGMA clustering method where a positive correlation of genetic variability with geographical distance among closely related populations could be clearly seen.

**Conclusion:**

The result aids in the identification, collection, and preservation of diverse germplasm of *I*. *griffithii* from Arunachal Pradesh and Meghalaya of Northeast India. This would further help in understanding the population structure and genetic diversity among other *Illicium* species in order to formulate effective conservation strategies for the improvement of this endangered taxa.

## Background

**Fig. 1:**
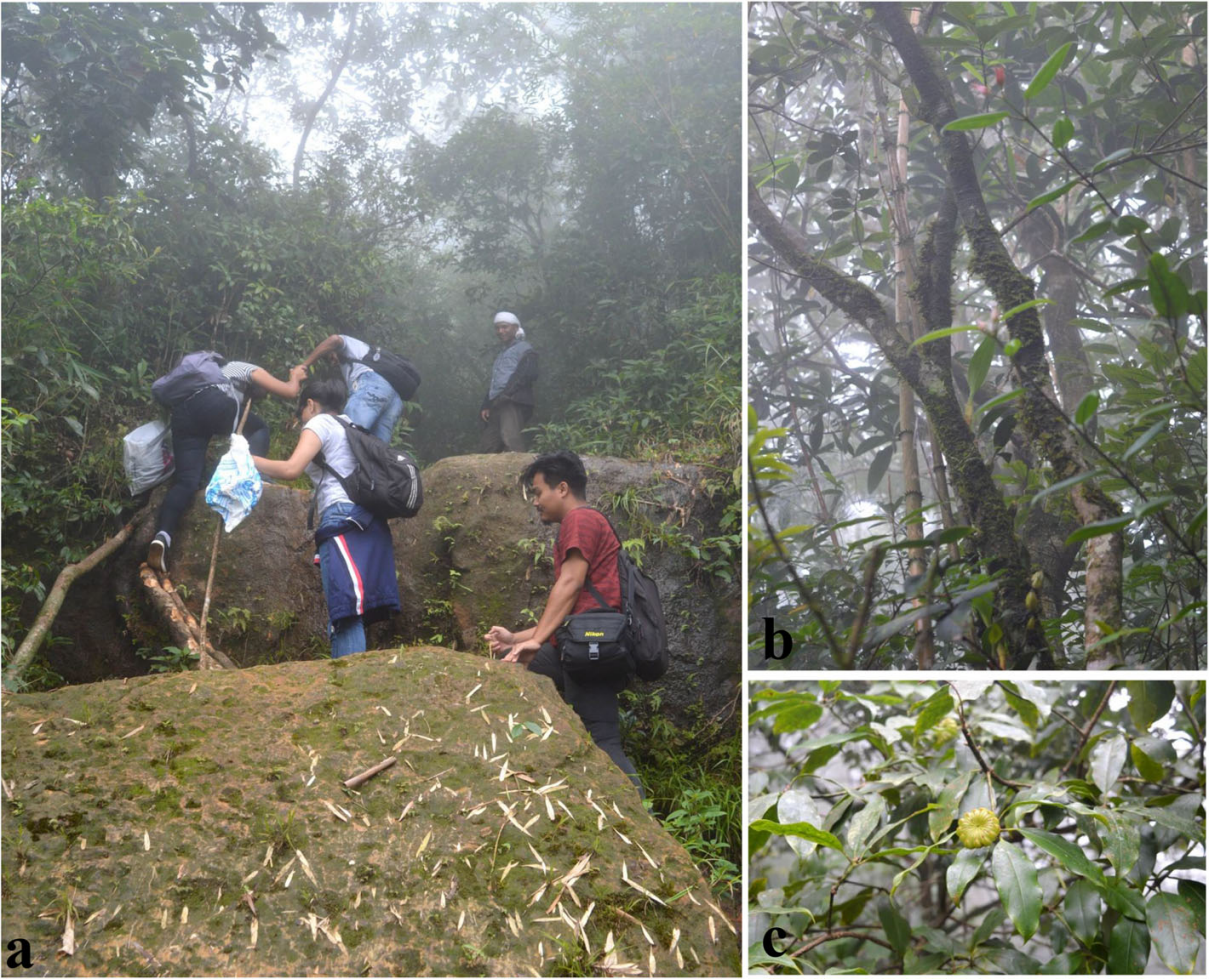
Collection of samples from different locations; **a** field collection, **b** *I*. *griffithii* plant, **c** *I*. *griffithii* plant bearing fruits

The Northeast Himalayan range in India is one of the major biodiversity hotspots that harbor numerous endemic and endangered species having medicinal and economic importance. The wide altitudinal and climatic variations favor the existence of different forest types creating a favorable niche to considerable number of medicinal plants and herbs in particular. The importance of medicinal plants was although overlooked in the past, presently they are valued as an affordable healthcare supply aiding to the economic growth of the country. According to a WHO report, over 80% of the world population relies upon plant based traditional medicines for primary healthcare [1]. However, medicinal plants which form the basis for modern drug discoveries around the world are a living and exhaustible resource if not used sustainably. Presently, with the increase in the market demand for modern drug manufacturing, the medicinal plants are exploited without concern for their regeneration and conservation. Due to over exploitation and various adverse anthropogenic factors, the forest cover is steadily shrinking and certain medicinal plants have become endangered [2]. Approximately 90% of the plant species used in the herbal industry is extracted from the wild and majority of these comes from the Himalayan region [3, 4]. Therefore, a large number of species are listed in IUCN Red Lists from this region. *Illicium griffithii* Hook. F. & Thoms. belonging to the family *Illiciaceae* is one such important aromatic and medicinal tree species that has been listed in the IUCN Red List (Fig. 1) [5].

*I*. *griffithii* is an evergreen tree distributed at an altitude of 1700–3000 m across the temperate and subtropical forests of Arunachal Pradesh, Manipur, Meghalaya, Nagaland, Bangladesh, Bhutan, and Myanmar [6]. However, within these places, it has a confined and restricted distribution and the species is found in selected forest patches with favorable microclimatic condition [7]. The flowers of *I*. *griffithii* are obligate xenogamous, and thus only compatible pollen grains germinate on the stigma. The flowers are brooding sites for the midges and the young ovules are eaten by the larvae of the midges. Only 10% of the flowers mature into 13-seeded fruits and the remaining 90% of the flowers have seeds ranging from 1 to 5 [8]. The seeds show considerable dormancy period due to hard seed coat [9] and the seedling survivability rate is only 7% [8]. The above factors adversely affect the natural regeneration of the species in the wild. Dried seed pods of *I*. *griffithii* have a good market potential in the spices and pharmaceutical industries. They are used for medicinal preparations that cure abdominal pain, cough, food poisoning, vomiting, toothache, etc. Fruits are the potential source of shikimic acid and used for the production of Tamiflu (oseltamivir) which is an active drug against avian influenza or bird flu [10]. Besides, it is used as an aromatic, carminative, stimulant, glactogogic, and antifungal agent. The fruits are also used in incense, flavoring agent, food preservative, and to enhance the potency of alcohol. Woods from mature trees are used as fuel, poles for construction of houses, fencing, etc. and the remarkable economic potential of the plant has favored an important natural off-income source for the rural people [7]. Besides the reproductive bottlenecks as stated above, in recent years, habitat degradation, unscientific harvesting, and rampant exploitation of the species to meet the raising market demands has caused a negative and adverse effect on its natural populations. *I*. *griffithii* with its poor regeneration in the wild, localized natural growth, high anthropogenic pressure, and intangible efforts for conservation have resulted the species to be categorized as critically endangered species in Meghalaya and endangered in Arunachal Pradesh [8, 11]. In order to conserve the gene pool, it is crucial to understand the genetic makeup of this important species. Proper knowledge of genetic diversity and population structure not only enhances our understanding of population dynamics, adaptation, and evolution of a particular species but also provides useful information for its conservation. Presently, various PCR-based molecular markers that are often based on non-coding DNA regions have proved beneficial to assess the genetic diversity among different plant species [12]. DNA-based molecular markers have various advantages over traditional methods and can effectively reveal the subtle variability at genetic levels with consistent data and authenticity. Polymerase chain reaction (PCR)-based single primer amplification reaction (SPAR) approach includes markers like random amplified polymorphic DNA (RAPD), inter-simple sequence repeats (ISSR), directed amplification of minisatellite DNA regions (DAMD), start codon targeted polymorphism (SCoT), SSR, etc., that collectively imparts an in-depth knowledge of the existing genetic diversity [13–18]. Since these markers are simple in nature, fast to perform, yield highly discriminative and reliable data, with a minimal cost involved, they are preferred by majority of plant biologists [19–22].

Previously, several studies have been carried out focusing on the biological factors causing the decline of *I*. *griffithii* population in the wild [8, 23–25]. But there is a large gap where genetic composition of *I*. *griffithii* that actually manifests in the form of morphological or reproductive variations concerning its survivability and evolution has been largely neglected. It was from this view point that the present study was undertaken so as to compare the various types of DNA based molecular markers namely RAPD, ISSR, DAMD, and SCoT by their efficiency and SPAR approach to evaluate the genetic diversity of *I*. *griffithii* as well as to analyze population genetic structure for conservation purpose.

## Methods

### Collection of plant materials

**Fig. 2:**
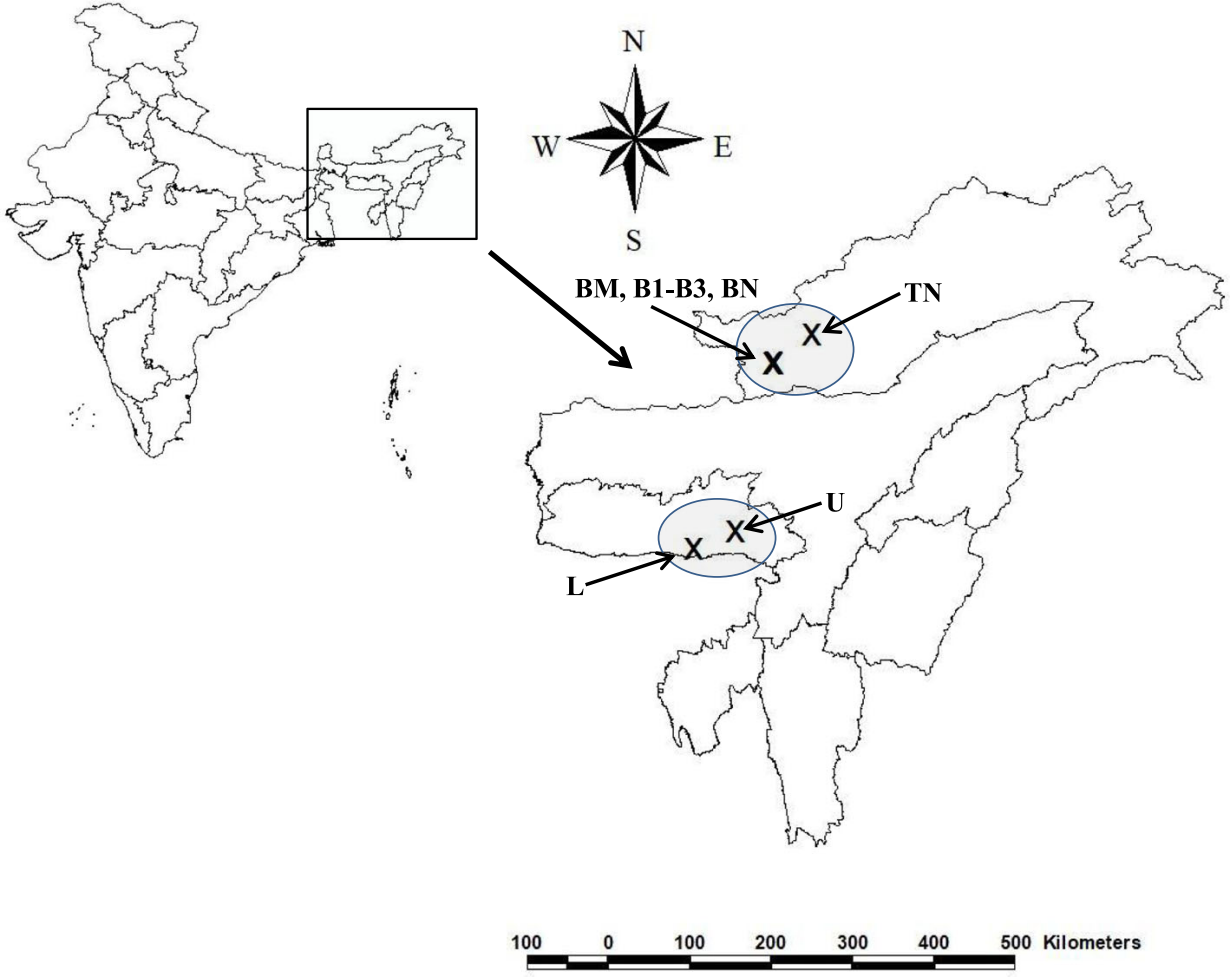
Geographical distribution of collected *I*. *griffithii* genotypes from Meghalaya and Arunachal Pradesh (x represents collection sites)

Plant materials for the study were collected from the natural habitats of Meghalaya and Arunachal Pradesh, India. Collected sites are tabulated below with their GPS readings (Table 1, Fig. 2). Young tender leaves (10–20) were randomly collected from each tree chosen at an interval of 30–50 m (a standard dependant on population size) according to the methods described by Roose et al. [26]. The sampling group included juvenile individuals of generative and pre-generative age (up to 10 m plant height). Sample size was maintained to a maximum of 30 individual per population from Arunachal Pradesh. However, in Meghalaya due to rarity of the species only a maximum of 10 individual per population could be maintained.

**Table. 1:** Collection sites of *I*. *griffithii* from different areas of Meghalaya and Arunachal Pradesh

Sl. No.	Sites	Population ID	Sample size	GPS reading	Elevation (meter)
1	Laitryngew Patch, Meghalaya	L	6	N 25°13′32.09′′ E 091°32′51.32′′	1558
2	Umtong Patch, Meghalaya	U	10	N 25°24′43.06′′ E 092°00′12.48′′	1507
3	Bomdila Monestry, A. Pradesh	BM	9	N 27°16′06.52′′ E 092°25′06.86′′	2526
4	Bomdila Patch 1, A. Pradesh	B1	18	N 27°16′31.16′′ E 092°25′34.28′′	2564
5	Bomdila Patch 2, A. Pradesh	B2	30	N 27°16′48.13′′ E 092°25′32.12′′	2496
6	Bomdila Patch 3, A. Pradesh	B3	30	N 27°16′36.63′′ E 092°25′33.43′′	2520
7	Bomdila Nursery, A. Pradesh	BN	6	N 27°16′39.15′′ E 092°25′35.94′′	2512
8	Tawang Nursery, A. Pradesh	TN	6	N 27°35′35.76′′ E 092°51′22.10′′	2850

### DNA extraction and SPAR

Genomic DNA was isolated from young leaves following the CTAB method [27] with minor modification. PCR was performed in 25 μL mixture containing approximately 40 ng DNA, 2.5 mM each of the four dNTPs, 10× PCR buffer (Mg^2+^ plus), 5 u/μL GoTaq DNA Polymerase (Takara), and 10 pmol of primer. Reactions were performed in a thermocycler (Eppendorf) with the settings as given in Table 2.

**Table. 2:** Reaction settings for the PCR cycles

Programme	Initial denaturation	PCR cycle			Final extension	No. of cycles
RAPD	94 °C (3 min)	94 °C (45 s)	36 °C (1 min)	72 °C (2 min)	72 °C (7 min)	45
ISSR	94 °C (3 min)	92 °C (2 min)	38–54 °C (1 min)	72 °C (2 min)	72 °C (7 min)	40
DAMD	94 °C (4 min)	94 °C (1 min)	50 °C (2 min)	72 °C (2 min)	72 °C (7 min)	40
SCoT	95 °C (5 min)	94 °C (45 s)	55 °C (1 min)	72 °C (2 min)	72 °C (7 min)	40

Amplified products were separated on 2% agarose gel stained with ethidium bromide in 1× TAE buffer and run for 3 h in an electrophoresis unit at 85 V. DNA bands were visualized and photographed in a gel documentation unit (Biostep DH-20, Germany) for final SPAR analysis. Each reaction was performed in two replicates and only clear reproducible bands were scored. Amplified products detected on the gels were recorded in a binary matrix where the fragments of similar size were represented as “1” for present or “0” for absent.

### Data analysis

Genetic similarity based on Jaccard’s coefficient was calculated using SIMQUAL module and a dendrogram was constructed using the NTSYS version 2.20 software package following the unweighted pair group method with arithmetic mean (UPGMA) option of the SAHN module [28]. As per the binary metrices, principal coordinate analysis (PCoA) was performed to correlate genetic relationships among the populations using PAST version 3.16 [29]. To estimate the level of genetic polymorphism, the percentage of polymorphic amplicons (Pp %), Shannon’s index (I), and Nei’s genetic diversity (the expected heterozygosity, H), the programme POPGENE version 1.31 was used [30]. Gene flow (Nm) was determined using the formula Nm = 0.25 × (1 − Gst) / Gst. The resolving capacity of the primers (Rp value) was calculated according to Prevost and Wilkinson [31].

To analyze the within and among population variations, analysis of molecular variance (AMOVA) at two hierarchical levels was performed using Arlequin version 3.01 [32]. F statistics (F_ST_) was applied to reveal differentiation between populations and its significance was evaluated following Wright [33]. Genetic population structure was investigated using Bayesian model-based clustering analysis with Structure 2.3.4 programme [34, 35] adapted to dominant markers. To determine the most likely number of groups (*K*) in the data, a series of analyses were performed from *K* = 1 through 11, using 25,000 burn-in and 25,000 repetitions, with 10 iterations per *K*.

## Results

The four marker systems (RAPD, ISSR, DAMD, and SCoT) used in the present study revealed significant amount of polymorphism independent of each other at various levels proving the usefulness of the SPAR system used, to analyze diversity studies in *I*. *griffithii*. In the present study, out of 100 primers that were initially screened, a total of 28 primers were finally selected for analysis.

### RAPD analysis

**Fig. 3:**
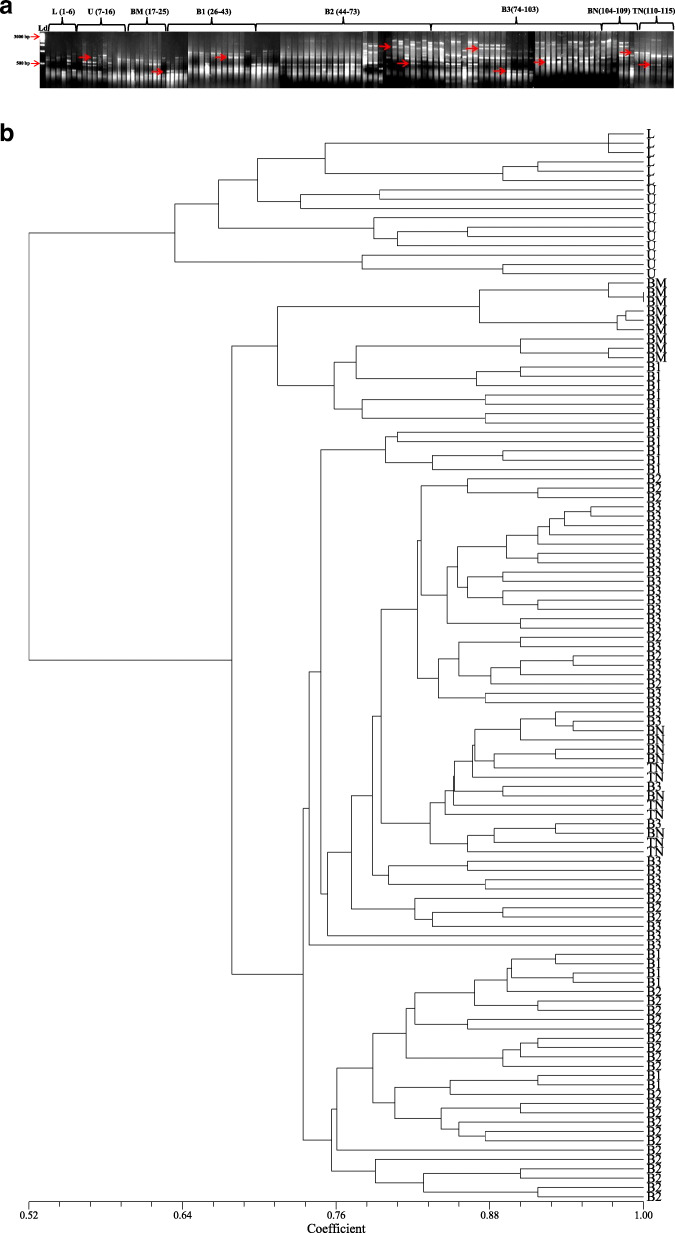
RAPD profiles; **a** banding profile in *I*. *griffithii* populations using primer OPK-4, **b** genetic distance recorded with RAPD primers alone

A total of 7 RAPD primers that were used for the analysis yielded 73 bands out of which 71 bands were polymorphic in nature (97.26%). The primer OPH-19 produced maximum band number (13) with a polymorphic information content (PIC) value of 0.76. The resolving power (Rp value) of RAPD primers ranged between 2.59 and 5.49. The amplicon size ranged between 0.2 and 2 kb with an average of 10.42 amplicons per primer (Table 4, Fig. 3a). The genetic distance recorded using Jaccard’s similarity coefficients ranged from 0.52 to 1.00 (Table 3, Fig. 3b).

**Table. 3:** Individual as well as collective comparison of SPAR methods (RAPD, ISSR, DAMD and SCoT)

Sl. No.	Name of SPAR approach	No. of primer used	Total bands amplified	Average bands/primer	Size of amplicons (range kb)	Total no. of polymorphic bands	% of polymorphism	Distance range (Jaccard’s coefficient)
1	RAPD	7	73	10.42	0.2–1.5	71	97.26	0.52–1.00
2	ISSR	7	61	8.71	0.2–2.0	60	98.36	0.59–1.00
3	DAMD	8	62	7.75	0.2–2.0	62	100.00	0.51–1.00
4	SCoT	6	54	9.00	0.2–2.0	53	98.14	0.55–1.00
5	RAPD + ISSR + DAMD + SCoT	28	250	8.92	0.2–2.0	246	98.40	0.57–1.00

### ISSR analysis

**Fig. 4:**
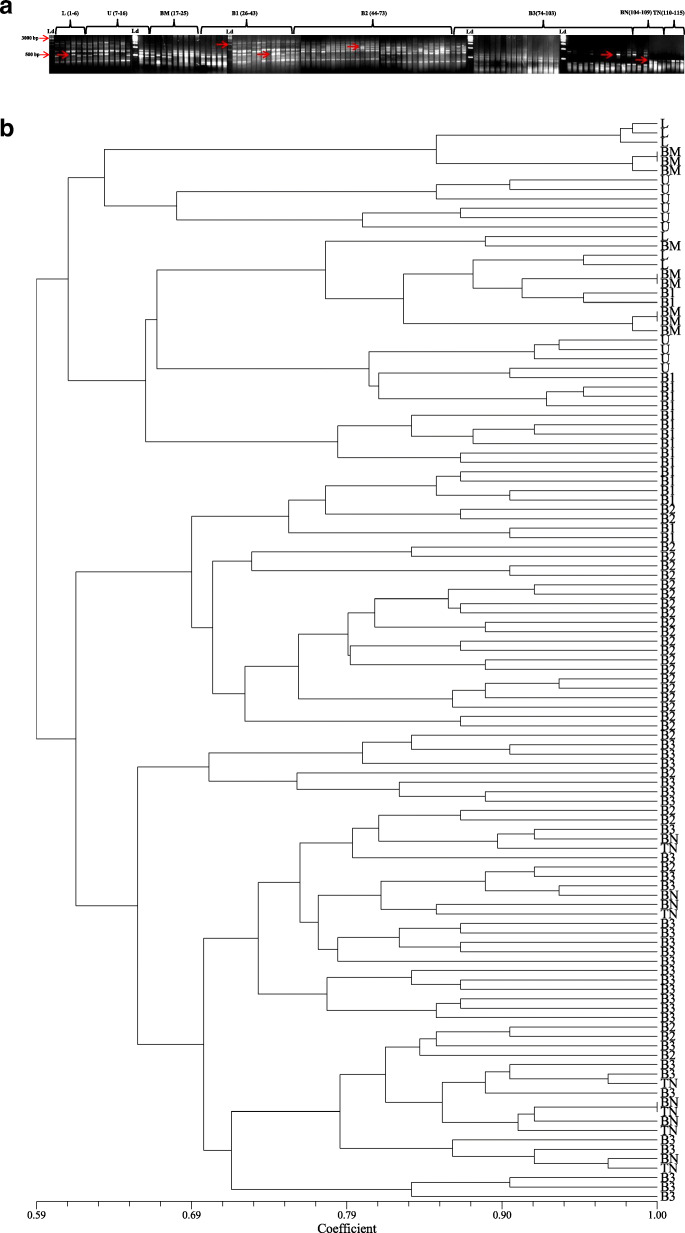
ISSR profiles; **a** banding profile in *I*. *griffithii* populations using primer SUNSRK-14, **b** genetic distance recorded with ISSR primers alone

There were 7 ISSR primers that were finally selected for genetic diversity analysis of *I*. *griffithii*. Out of 61 bands that were generated, 60 bands were found polymorphic (98.36%). The primer SUNSRK-6 produced maximum band number (12) with a PIC value of 0.88. The resolving power (Rp value) of the primers ranged between 2.66 and 6.34. The amplicon size ranged between 0.2 and 2 kb with an average of 8.71 amplicons per primer (Table 4, Fig. 4a). The genetic distance recorded using Jaccard’s similarity coefficients ranged from 0.59 to 1.00 (Table 3, Fig. 4b).

**Table. 4:** Extent of polymorphism as revealed by RAPD, ISSR, DAMD and SCoT primers

Sl. No.	Name of primer	Primer’s sequence	Total no. of band	No. of poly-morphic band	No. of mono-morphic band	% of poly-morphic bands	Resolv-ing power (Rp)	PIC (Poly-morphic information content)	Distance range (Jaccard's coefficient)
RAPD									
1	OPA-11	5′-CAATCGCCGT-3′	9	8	1	88.88	4.60	0.88	0.57-1.00
2	OPA-12	5′-TCGGCGATAG-3′	12	12	0	100.00	5.49	0.83	
3	OPA-13	5′-CAGCACCCAC-3′	11	11	0	100.00	5.00	0.87	
4	OPB-1	5′-GTTTCGCTCC-3′	11	11	0	100.00	4.64	0.80	
5	OPB-6	5′-TGCTCTGCCC-3′	10	9	1	90.00	3,75	0.79	
6	OPH-19	5′-CTGACCAGCC-3′	13	13	0	100.00	5.32	0.76	
7	OPK-4	5′-CCGCCCAAAC-3′	7	7	0	100.00	2.59	0.70	
ISSR									
8	SUNSRK-5	5′-GAGAGAGAGAGAGAGATT-3′	9	9	0	100.00	3.93	0.86	
9	SUNSRK-6	5′-CTCTCTCTCTCTCTCTTA-3′	12	12	0	100.00	6.03	0.88	
10	SUNSRK-11	5′-GGCGGCGGCGGCGGCGGC-3′	8	8	0	100.00	4.45	0.89	
11	SUNSRK-14	5′-CACACACACACAAC-3′	10	10	0	100.00	6.34	0.88	
12	SUNSRK-14	5′-CACACACACACAGG-3′	7	7	0	100.00	5.77	0.83	
13	SUNSRK-21	5′-ACACACACACACACACAG-3′	8	8	0	100.00	4.95	0.74	
14	SUNSRK-22	5′-ACACACACACACACACT-3′	7	6	1	85.71	2.66	0.70	
DAMD									
15	URP 13R	5′-TACACGTCTCGATCTACA-3′	5	5	0	100.00	2.45	0.87	
16	Oligo 5	5′-GACNGGNACNGG-3′	10	10	0	100.00	2.97	0.95	
17	URP 38F	5′-AAGAGGCATTCTACCACC-3′	6	6	0	100.00	3.40	0.88	
18	URP 9R	5′-ATGTGTGCGATCAGTTGC-3′	7	7	0	100.00	4.57	0.79	
19	HBV 3	5′-GGTGAAGCSCAGGTG-3′	9	9	0	100.00	3.94	0.73	
20	URP 25F	5′-GATGTGTTCTTGGAGCCT-3′	8	8	0	100.00	4.53	0.79	
21	Oligo 2	5′-CTCTGGGTGTCGTGC-3′	9	9	0	100.00	5.60	0.69	
22	HVY	5′-GCCTTTCCCGAG-3′	8	8	0	100.00	4.33	0.69	
SCoT									
23	SCOT-1	5′-CAACAATGGCTACCACCA-3′	8	8	0	100.00	3.56	0.84	
24	SCOT-2	5′-CAACAATGGCTACCACCC-3′	8	8	0	100.00	3.30	0.90	
25	SCOT-13	5′-ACGACATGGCGACCATCG-3′	8	8	0	100.00	4.57	0.86	
26	SCOT-14	5′-ACGACATGGCGACCACGC-3′	10	10	0	100.00	4.41	0.84	
27	SCOT-22	5′-AACCATGGCTACCACCAC-3′	10	10	0	100.00	4.57	0.80	
28	SCOT-23	5′-CACCATGGCTACCACCAG-3′	10	9	1	90.00	3.40	0.76	
		Total	250	246	4	98.40			
		Average	08.92	08.78	0.14

### DAMD analysis

**Fig. 5:**
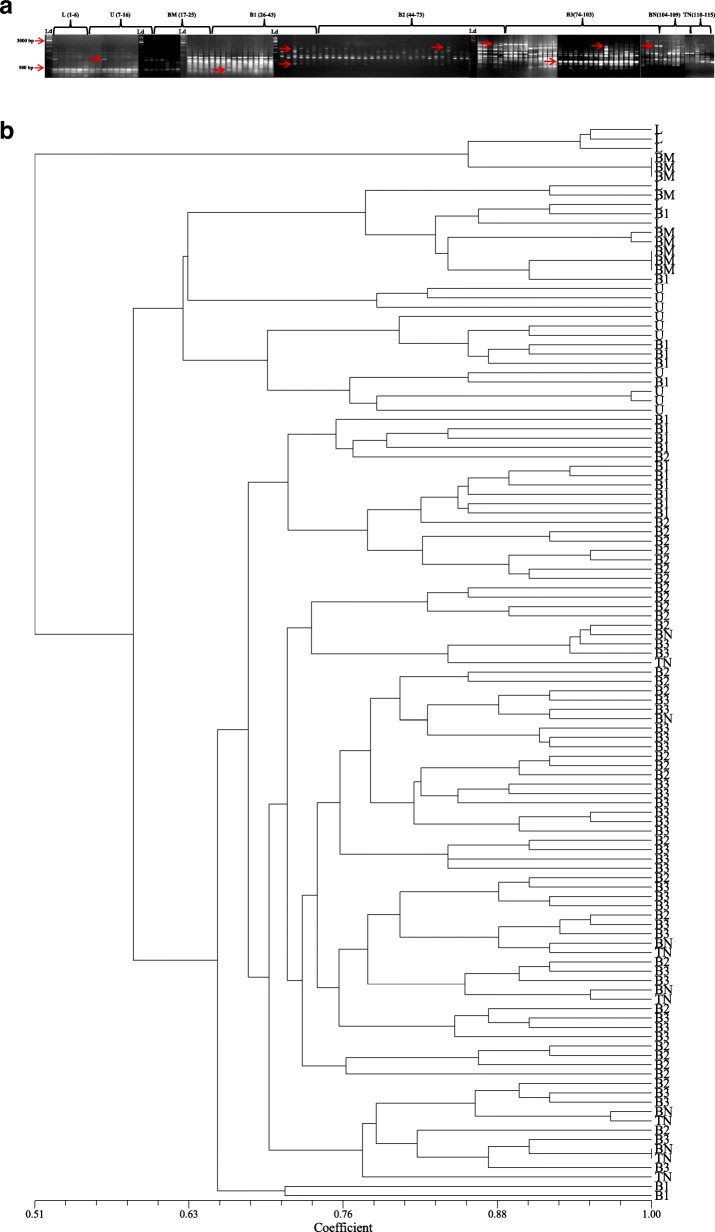
DAMD profiles; **a** Banding profile in *I*. *griffithii* populations using URP 38F, **b** genetic distance recorded with DAMD primers alone

A total of 8 DAMD primers were finally selected for the analysis. The primers yielded 62 bands which were all polymorphic in nature (100%). The primer Oligo 5 produced maximum band number (10) with highest polymorphic information content (PIC) of 0.95. The resolving power (Rp value) of the primers ranged between 2.45 and 5.60. The amplicon size ranged between 0.2 and 2 kb with an average of 7.75 amplicons per primer (Table 4, Fig. 5a). The genetic distance recorded using Jaccard’s similarity coefficients ranged from 0.51 to 1.00 (Table 3, Fig. 5b).

### SCoT analysis

**Fig. 6:**
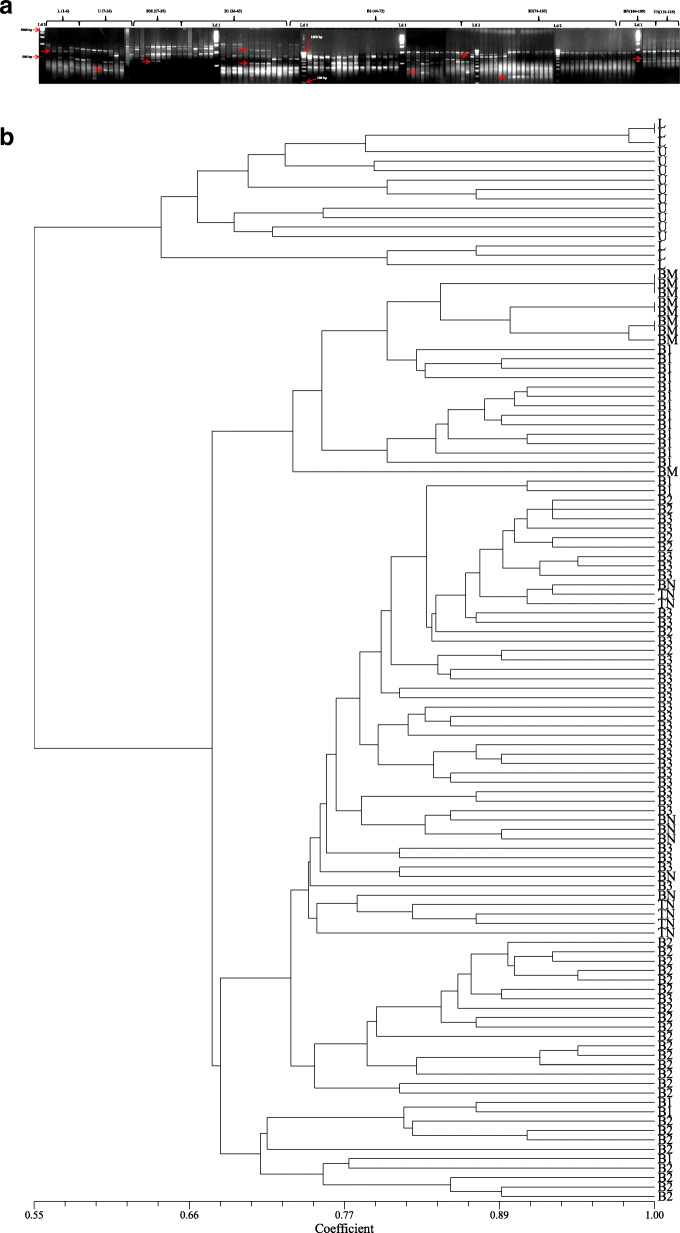
SCoT profiles; **a** Banding profile in *I. griffithii* populations using primer SCOT-1, **b** Genetic distance recorded with SCoT primers alone

Six SCoT primers were selected for analysis that produced a total of 54 bands out of which 53 bands were polymorphic (98.14%). Three SCoT primers (namely SCoT 14, 22, and 23) yielded maximum bands numbers (10) with PIC ranging between 0.76 and 0.84. The resolving power (Rp value) of the SCoT primers ranged between 3.30 and 4.57. The amplicon size ranged between 0.2 and 1.5 kb with an average of 9 amplicons per primer (Table 4, Fig. 6a). The genetic distance recorded using Jaccard's similarity coefficients ranged from 0.55 to 1.00 (Table 3, Fig. 6b).

### Combined study of RAPD, ISSR, DAMD, and SCoT (SPAR analysis)

To test the efficacy of the single primer-based amplification reactions commonly known as SPAR, the combined study of the above-mentioned primers (namely RAPD, ISSR, DAMD, and SCoT) was performed using the cumulative dataset in order to give a holistic approach to the study. The study yielded 250 amplicons out of which 246 (98.40 %) were polymorphic in nature (Table 3). The mean value of PIC (polymorphic information content) was found to be 0.81 with an Rp value (resolving power) of 4.32 (Table 4). The amplicon size ranged between 0.2 and 2.0 kb with an average of 8.92 amplicons per primer. The genetic distance recorded using Jaccard’s similarity coefficients ranged from 0.57 to 1.00 (Table 3).

### Population structure

The Pp% (percentage of polymorphic loci) for a single population ranged from 41.20% (BN) to 76% (B2) with a total value of 98.40%. The samples collected from B2 region have the highest observed number of alleles (Na) being (1.76 ± 0.42) while that of BN (1.41 ± 0.49) have the lowest (Na) (Table 5). For all the populations, the effective number of alleles (Ne) were consistently less than Na values showing a variation in the range of 1.26 (BN) to 1.46 (U) with an average of 1.55 ± 0.30. Population from U and B1 showed the highest Nei’s gene diversity (H) and Shannon index (I) (H = 0.26 ± 0.19; *I* = 0.39 ± 0.27) with the lowest being recorded in BN (Table 5). Mean coefficient of genetic differentiation among the eight sampled populations (Gst, assuming Hardy-Weinberg Equilibrium) was 0.396, showing that a higher level of population differentiation was distributed within populations.

**Table. 5:** Genetic variations as revealed through combined SPAR approach among eight populations of *I*. *griffithii*

Population	***N***	Na ± SD	Ne ±SD	H ± SD	I ± SD	Pp (%)	Np	Hsp	Hpop	Gst	Nm
L	06	1.48 **±** 0.50	1.38 **±** 0.43	0.20 **±** 0.22	0.29 **±** 0.31	48.00	120				
U	10	1.66 **±** 0.47	1.46 **±** 0.39	0.26 **±** 0.20	0.38 **±** 0.29	66.00	165				
BM	09	1.44 **±** 0.49	1.31 **±** 0.38	0.17 **±** 0.21	0.25 **±** 0.30	44.40	111				
B1	18	1.73 **±** 0.44	1.44 **±** 0.36	0.26 **±** 0.19	0.39 **±** 0.27	73.20	183				
B2	30	1.76 **±** 0.42	1.40 **±** 0.33	0.24 **±** 0.17	0.37 **±** 0.25	76.00	190				
B3	30	1.70 **±** 0.45	1.36 **±** 0.34	0.22 **±** 0.18	0.34 **±** 0.26	70.00	175				
BN	06	1.41 **±** 0.49	1.26 **±** 0.35	0.15 **±** 0.19	0.23 **±** 0.28	41.20	103				
TN	06	1.43 **±** 0.49	1.27 **±** 0.36	0.16 **±** 0.19	0.24 **±** 0.28	43.20	108				
Total	115	1.98 **±** 0.12	1.55 **±** 0.30	0.33 **±** 0.13	0.50 **±** 0.17	98.40	246	0.352	0.212	0.396	0.761

*N* sample size, *Na* observed no. of alleles, *Ne* effective no. of alleles, *H* Nei’s genetic diversity, *I* Shannon’s information index, *Pp* percentage of polymorphic loci, *Np* number of polymorphic loci, *Hsp* total variability, *Hpop* variability within population, *Gst* diversity among populations, *Nm* gene flow 0.5 (1 – Gst) / Gst, *SD* standard deviation

This was validated with AMOVA analysis where higher variation (69.37%) within populations as compared to lower variation (30.63%) among populations was recorded with an F statistics (FST = 0.30) (Table 6). The overall level of inferred gene flow (Nm) was estimated at 0.761, showing a relatively low migration rate between populations. Table 7 represents Nei’s unbiased measure of genetic identity with genetic distance among the collected populations.

**Table. 6:** Analysis of molecular variance (AMOVA) showing variation in the collected populations of *I*. *griffithii*

Source of variation	Degrees of freedom	Sum of squares	Variance component	Percentage of variation	F_**ST**_
Among populations	07	1490.544	13.56	30.63	
Within populations	107	3287.578	30.72	69.37	
Total	114	4778.122	44.29	100	0.30

**Table. 7:** Nei’s unbiased measures of genetic identity and genetic distance among *I*. *griffithii* populations

Population ID	L	U	BM	B1	B2	B3	BN	TN
L	****	0.8355	0.7770	0.7388	0.6949	0.6868	0.6546	0.6574
U	0.1797	****	0.7644	0.7951	0.7535	0.7513	0.7048	0.7017
BM	0.2523	0.2687	****	0.8532	0.7739	0.7660	0.7293	0.7315
B1	0.3028	0.2293	0.1588	****	0.9191	0.8758	0.8387	0.8289
B2	0.3641	0.2830	0.2563	0.0844	****	0.9372	0.8831	0.8691
B3	0.3757	0.2859	0.2666	0.1326	0.0648	****	0.9344	0.9122
BN	0.4238	0.3498	0.3156	0.1760	0.1243	0.0679	****	0.9677
TN	0.4194	0.3543	0.3126	0.1877	0.1403	0.0919	0.0328	****

Nei’s genetic distance (below diagonal) and genetic identity (above diagonal)

**** Signify zero distance since genetic distance is calculated within the population itself (e. g. between L and L = zero distance)

### Cluster/Tree analysis

**Fig. 7:**
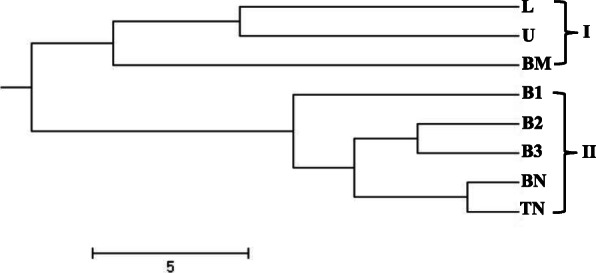
UPGMA clustering of *I*. *griffithii* populations based on Jaccard's similarity

**Fig. 8:**
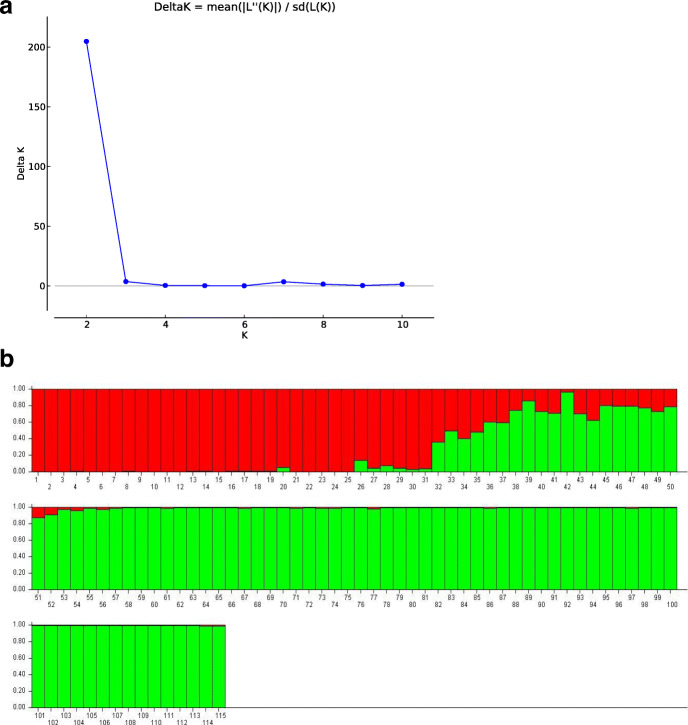
Structure analysis based on Bayesian clustering of 8 populations (115 accessions); **a** plot showing the ΔK values; **b** genetic clustering estimated (*K* = 2) showing two genetic pools

**Fig. 9:**
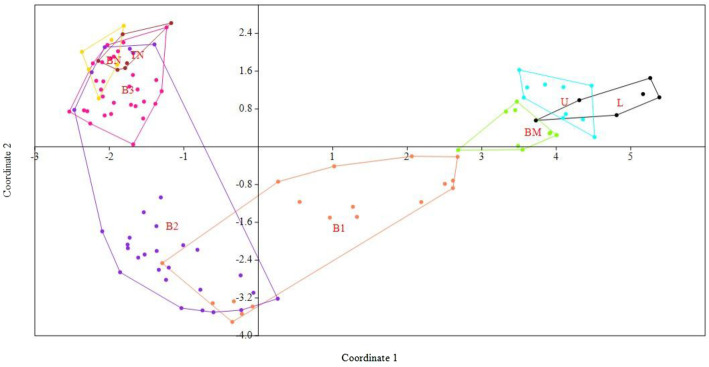
Principal coordinate analysis (PCoA) revealing the clustering pattern of 8 populations (115 accessions) of *I*. *griffithii*

A dendrogram was constructed from the compiled data set of RAPD, ISSR, DAMD, and SCoT to represent the relationships among the collected populations using the UPGMA method of SAHN clustering. The populations under study were separated into two distinct clusters (Fig. 7). Cluster-I was again sub-clustered into Ia comprising population from L and U (Meghalaya) and Ib having population from BM (Arunachal Pradesh). Cluster-II further sub-clustered into IIa (with B1), IIb (B2 and B3), and IIc (BN and TN) all from Arunachal Pradesh population (Fig. 7). Bayesian clustering using structure analysis also revealed congruent results where the optimal ΔK for *K* = 2 is the best fit model to group the populations into two clusters (Fig. 8). The PCoA result derived using PAST showed similar clustering pattern that was consistent with the UPGMA and Bayesian clustering results generated in the study (Fig. 9).

## Discussion

The assessment of the level and distribution of genetic variability in the wild plant species provides essential information regarding its evolutionary history and has a pivotal role in the conservation and maintenance of genetic resources [36–39]. Several important aspects of conservation biology concerning the loss of genetic diversity and restoration of threatened or endangered populations can only be addressed through detailed population genetics studies [40]. DNA-based molecular marker approaches are far more advanced over traditional methods with its high authenticity and consistency that can reveal even subtle genetic variability at DNA levels. However, the choice of techniques and proper markers are debatable and mostly depend on the nature of genetic structure of the species. The rate of evolutionary changes for a particular genomic region may differ according to species and therefore, it requires a distinctive approach to target various genomic areas as potential molecular genetic markers [41]. In this context, a comparison is required so as to decide which approach is most suitable for the taxon under study [42]. The present study deals with the collective use of four marker system (RAPD, ISSR, DAMD, and SCoT) to examine the extent of genetic variability among the natural populations of *I*. *griffithii* collected from Meghalaya and Arunachal Pradesh.

It was found that each marker system was capable of detecting significant genetic polymorphism among the *I*. *griffithii* populations proving its discriminating efficacy and applicability in the current study. DAMD markers were found to be the most effective with 100% polymorphic bands (Table 4). The range of Rp values and PIC content obtained with all other markers were also adequate confirming their genotype discriminating ability [31, 43]. However, a marker is only considered best when there is a fine focus on the repetitive sequences of a particular genome, including *I*. *griffithii*. Therefore, a combination of RAPDs (spanning the entire genome of the DNA), ISSRs/DAMDs (spanning selected repetitive sequences), and SCoTs (spanning the start codon regions) could be considered as suitable markers for more meaningful and holistic approach to the analysis of genetic variability. Several authors have stressed the advantages of using more than one class of molecular markers to estimate genetic diversity of threatened taxa [44, 45]. Numerous reports are available where SPAR approach has been used extensively to understand the intra- as well as inter-level population variation among various plant species [21, 46–49]. In the present analysis, 28 SPAR markers used were capable of detecting a high level of genetic variation at the species level with 98.40% of bands being polymorphic (Table 4). The range of values that determine the genome composition in a population like allelic frequency (Na = observed no. of alleles and Ne = effective number of alleles), gene flow (Nm), and Nei’s genetic differentiation (Gst) varied significantly than the corresponding values reported earlier [50–53]. This may be attributed to the inherent genotypic differences or the combination of marker system used for analysis.

The value of Nei’s genetic differentiation among populations (Gst) may range from zero to one, with a higher value indicating that a larger number of variations lie among various populations. In the present study, different methods (Nei’s, Gst, and AMOVA analysis) showed similar values of genetic differentiation among populations (Gst = 0.396, F_ST_ = 0.30, respectively), revealing a low level of genetic differentiation among the eight sampled populations of *I*. *griffithii* (Tables 5 and 6). Most of the out-crossing species, in particular, usually follow this pattern where higher levels of genetic diversity resides within populations and low genetic diversity is found among populations [40, 54, 55]. Also, it has been confirmed that in nature gene flow can be estimated as low (with Nm less than 1), moderate (Nm greater than 1), and extensive (Nm greater than 4) as reported by Kumar et al. [22]. *I*. *griffithii* with an estimated gene flow value of Nm = 0.761 was significantly low among populations which is a characteristic feature of rare and endangered species [56]. *I*. *griffithii* shows obligate xenogamy and the breeding system is mostly entomophilous (insects facilitated cross pollination) [25]. Insects have limited abilities to fly, and seed or pollen dispersal separated by long distances (as far as 25–100 km.) is an unlikely or rare event. The lower level of gene flow among populations (Nm lesser than 1) as seen in the present case is either due to population isolation caused by habitat fragmentation (natural or man-made) and/or lack of long-distance seed dispersers or pollinators (as the hilly terrain together with fragmented population restricts normal pollen transfer for short distant flyers). Our result conforms to the study of Duchok et al. [57] where they have considered the population pattern and regeneration ability of *I. griffithii* across disturbed area of Arunachal Pradesh. The study revealed clear dominance of adult trees and lowest number of seedlings across the study sites indicating poor regeneration of *I*. *griffithii*. Fruits on maturity expel their seeds to nearby areas which although are capable of germinating fails to convert into early sapling stage justifying the negative role of disturbance that creates an adverse niche for regeneration of *I*. *griffithii* [57]. Few seedlings that survive, again results in local regeneration favoring maximum variation retained within population. Clustering pattern obtained with Bayesian structure (Fig. 8) and PCoA diagram (Fig. 9) is also indicative of similar results and revealed that intermixing of genetic material across populations is only possible when the populations lie close to each other. This is further validated with UPGMA clustering method where a positive correlation of genetic variability with geographical distance among closely related populations could be clearly seen (Fig. 7).

Among the eight populations sampled here, BM from Arunachal Pradesh showed relatively lower levels of genetic variation. This is due to its peripheral distribution range from the collected central populations (B1, B2, and B3) (Table 1). Populations located at the margins that are separated spatially from central populations are often believed to have smaller population sizes and lower genetic variations [58–60]. In addition, smaller sample size in population L (6), U (10) from Meghalaya, and BN (6) and TN (6) from Arunachal Pradesh could contribute to the lower genetic variation of the populations. From our field survey and ethno-botanic investigation, it was also observed that over collection of *I*. *griffithii* fruits to meet the increasing demands have negatively affected its natural population. Overharvesting of its wild resources has resulted in a sharp decline of the effective population number and size, with patches of local disappearance. Numerous studies are available which confirm the loss of genetic variation in wild populations due to overharvesting adversely impacting the evolutionary potential of a species to adapt to changing environments [61, 62]. Therefore, the observed pattern of genetic differentiation among the populations of *I*. *griffithii* may be attributed to all or some of the aforesaid factors mentioned in the study. Our results corroborate with the findings of other workers where moderate genetic differentiation among populations was observed in contrast to maximum genetic variability retained within populations [63–67].

## Conclusion

Scientific approaches for conservation and sustainable utilization of plant resources require accurate assessment of the amount and distribution of genetic variation within and among populations [68]. Molecular marker techniques are reliable and far more advanced over traditional method to gain insight into the subtle genetic diversity affecting plant population structure [69, 70]. The present study showed the effectiveness of SPAR markers for the analysis of substantial genetic variability affecting genetic structure within natural populations of *I*. *griffithii*. The high genetic diversity within populations indicates that the best method for conservation would be to protect the existing natural populations and reintroduction of more individuals into the affected areas to maintain maximum diversity. Alternative techniques like macropropagation (cutting, grafting, etc.) and micropropagation (and/or in vitro seed germination) can be explored to effectively raise individuals from different populations and reintroduce them into the wild. The present study aids in identification, collection, and prioritization of genetically diverse germplasms of the threatened taxon *I*. *griffithii* for improvement and conservation.

## Data Availability

The data sets used and analyzed in the present study are presented in the article and the voucher specimen deposited in Botanical Survey of India (BSI), Eastern Regional Centre, Shillong, Meghalaya, India with an accession number.

## Abbreviation

PIC: Polymorphic information content
Rp: Resolving power
RAPD: Random amplified polymorphic DNA
AFLP: Amplified fragment length polymorphism
DAMD: Directed amplification of minisatellite DNA regions
ISSR: Inter-simple sequence repeats
SCoT: Start codon targeted polymorphism
SPAR: Single primer amplification reaction
Pp: Percentage of polymorphic amplicons
I: Shannon’s index
H: Nei’s genetic diversity
Nm: Gene flow
F_ST_: Fixation index or F statistics
UPGMA: Unweighted pair group method with arithmetic mean
PCoA: Principal coordinate analysis
